# Factors Predicting the Final Diagnosis in Image-Guided Percutaneous Needle Biopsy for Suspected Spinal Tumors

**DOI:** 10.3390/jcm12134292

**Published:** 2023-06-26

**Authors:** Makoto Oka, Akinobu Suzuki, Hidetomi Terai, Minori Kato, Hiromitsu Toyoda, Shinji Takahashi, Koji Tamai, Hiroaki Nakamura

**Affiliations:** Department of Orthopaedic Surgery, Graduate School of Medicine, Osaka Metropolitan University Osaka, Osaka 545-8585, Japan; ako3otokam1@gmail.com (M.O.); terai@omu.ac.jp (H.T.); minori202048@gmail.com (M.K.); h-toyoda@omu.ac.jp (H.T.); a99m042@yahoo.co.jp (S.T.); koji.tamai.707@gmail.com (K.T.); hnakamura@omu.ac.jp (H.N.)

**Keywords:** spine, tumor, needle biopsy, metastasis, diagnosis, malignant history, computed tomography, magnetic resonance image

## Abstract

In cases of suspected spinal tumors on imaging studies, a biopsy is often necessary for establishing the diagnosis. Predictive factors for tumors or malignancies may help in scheduling biopsies or avoiding unnecessary ones. However, there have been few studies on determining these factors. We aimed to determine the factors associated with the final diagnosis in cases requiring spinal biopsy. This study included 117 patients who underwent image-guided (fluoroscopy- or computed tomography [CT]-guided) needle biopsy of the spine. Data on patient demographic, pathological diagnoses, and final diagnoses were retrospectively collected from the medical records. The imaging features and location of lesions were also evaluated on CT and magnetic resonance imaging. Furthermore, factors related to tumors or malignancies were analyzed. The diagnostic accuracy of biopsy was 94.0%, and there was no difference in the diagnostic accuracy between the fluoroscopic and CT-guided biopsies. Sixty-six and fifty-six patients were diagnosed with spinal tumors and malignant tumors, respectively. Multivariate analysis revealed that a history of malignant tumors and the presence of pedicle lesions and/or extravertebral lesions were related factors for both tumors or malignancy in the final diagnosis. These findings can help determine the necessity for or timing of biopsy in patients with suspected spinal tumors.

## 1. Introduction

Recent developments in treatment have improved prognosis in patients with malignant tumors, and the number of patients surviving metastases is increasing [1]. Bone metastasis is common in patients with malignant tumors. Autopsy studies have reported bone metastasis in 70% of patients diagnosed with cancer [2]. The spine is the most common site of bone metastases [3]. Spinal metastases cause axial pain, radicular pain, and paralysis due to pathological fractures and/or spinal cord compression, resulting in severe impairment of quality of life [4,5,6].

Diagnosis of spinal metastases is important for the prevention and treatment of these events. Diagnosis is often based on a history of malignancy and imaging studies, such as computed tomography (CT) and magnetic resonance imaging (MRI). However, it is often difficult to determine whether an imaging abnormality is a tumor and whether it is a primary or metastatic tumor, particularly in patients without a history of malignancy or those who have completed treatment in an early stage of the disease. Furthermore, in patients with a history of multiple malignancies, identifying the primary tumor is difficult. In such cases, a biopsy is necessary to confirm the diagnosis and determine the treatment plan. However, a biopsy is an invasive procedure. Furthermore, the biopsy results may show non-neoplastic tissue, such as post-fracture vertebral changes or hyperplastic bone marrow. In addition, follow-up imaging examination rather than biopsy may be sufficient for identifying those non-tumoral pathologies. Predicting the final diagnosis based on patient data and/or imaging findings could help determine the necessity or urgency of a biopsy. However, few studies have assessed these factors. This study aimed to investigate the factors associated with the final diagnosis in patients who underwent image-guided percutaneous needle biopsy for suspected tumors that were difficult to diagnose.

## 2. Materials and Methods

Patients who underwent percutaneous needle biopsy by skilled spine surgeons between 2013 and 2022, owing to suspicion of a spinal tumor based on imaging studies and difficulty in diagnosis, were included in this study. Cases of intradural spinal cord tumors, such as intradural extramedullary and intramedullary tumors, were excluded from the study. Patients in whom metastasis from the primary tumor was clearly suspected did not undergo biopsy and were also excluded from this study. Cases in which biopsies were performed for culture due to suspected infection or insufficient CT or MRI data were also excluded. All methods were performed in accordance with the Declaration of Helsinki and Ethical Guidelines for Medical and Health Research Involving Human Subjects in Japan. The study protocol was approved by our Institutional Review Board (no. 3170).

**Figure 1:**
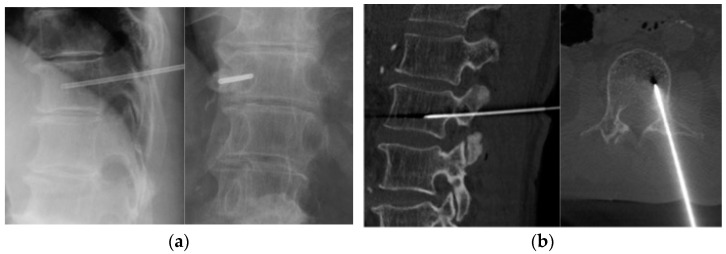
Image-guided percutaneous needle biopsy. (**a**) Fluoroscopic biopsy. (**b**) CT-guided biopsy.

Finally, 117 patients (mean age of 62.5 years; 73 males) were included in this retrospective study (Table 1). All biopsies were performed under image guidance with local anesthesia. Thoracic, lumbar, and larger lesions were assessed under fluoroscopy (73 cases), whereas cervical, sacral, and smaller lesions were assessed under CT guidance (44 cases) (Figure 1). The most common vertebral level was the lumbar spine in 57 patients (48.7%), followed by the thoracic spine in 37 (31.6%), sacral spine in 16 (13.7%), and cervical spine in 7 patients (6.0%).

All study variables and imaging data were retrospectively collected from the electronic medical records. The patient data included age, sex, history of malignancy, pathological diagnosis, and final diagnosis. Imaging findings were evaluated using the images examined on the date closest to the biopsy by comparing the area around the biopsy location with these of the other vertebrae that appeared normal. The findings of lesions on CT images were categorized into 4 groups according to the density change in the lesion: (i) osteolytic change, (ii) osteoblastic change, (iii) mixed change, and (iv) no change. The change in signal intensity of the lesion was also evaluated on T1- and T2-weighted MRI scans. The location of the lesions was comprehensively evaluated using CT and MRI to determine the presence or absence of the vertebral body, pedicle, posterior element, and extravertebral area.

The percentage of pathological and final diagnoses that were concordant was defined as the positive diagnosis accuracy. The diagnostic accuracy between the groups that underwent fluoroscopic and CT-guided biopsies were statistically compared using Fisher’s exact test. 

Based on the final diagnosis, all patients were classified into (1) tumor and non-tumor groups and (2) malignancy and non-malignancy groups. Univariate analyses were performed for age; sex; history of malignancy; imaging findings (CT and MRI); and presence of vertebral body, pedicle, posterior element, and extravertebral lesions. Continuous variables were analyzed using the Mann–Whitney U test. Categorical variables were analyzed using Fisher’s exact test or the chi-square test. Multivariate logistic regression analysis was performed using factors with *p* < 0.1 from univariate analysis, and explanatory variables, such as age, sex, and a history of malignancy, which were considered clinically important, and odds ratios (ORs) with 95% confidence intervals were calculated. All statistical analyses were performed using R software (version 3.5.1, Patched, http://www.r-project.org accessed on 12 January 2023; R Core Team, Vienna, Austria). Statistical significance was set at *p* < 0.05.

## 3. Results

Of the 117 patients included, 110 (94.0%) had a pathological diagnosis consistent with the final diagnosis based on percutaneous needle biopsy. Although three patients had no tumor tissue on pathology, they were finally diagnosed with tumors using other examinations. In the other four patients, the histological diagnosis could not be made because of insufficient quality/quantity of the tissue obtained by biopsy. Finally, 66 patients (56.4%) were diagnosed with either benign or malignant tumors, and 56 (47.9%) were diagnosed with malignant tumors. Among 43 patients with a history of malignancy, only 17 (39.5%) were diagnosed with the same malignant tumor as the previously diagnosed one (Table 2). There was no significant difference in the diagnostic accuracy between the fluoroscopic (93.2%) and CT-guided biopsies (95.5%) (*p* = 0.709) (Table 3). There was no complication of the biopsy procedure in both groups.

On CT images, osteolytic changes were the most common (52 cases, 44.4%), followed by no changes (34 cases, 29.1%), osteoblastic changes (21 cases, 17.9%), and mixed changes (10 cases, 8.5%). T1-weighted MR images showed low signal intensity in 111 cases (94.9%), high intensity in 4 cases (3.4%), and mixed intensity in 2 cases (1.1%). On T2-weighted MR images, 70 (59.8%) patients showed low signal intensity, 29 (24.8%) exhibited high intensity, and 18 (15.4%) showed mixed intensity. Vertebral body lesions were observed in 111 (94.9%) patients, pedicle lesions in 76 (65.0%), posterior element lesions in 43 (36.8%), and extravertebral lesions in 61 (52.1%) (Table 4).

Comparing each factor between the tumor and non-tumor groups, univariate analysis showed significant differences in CT imaging (*p* < 0.01), pedicle lesions (*p* < 0.01), posterior element lesions (*p* < 0.01), and extravertebral lesions (*p* < 0.01) (Table 5). On CT images, osteolytic changes were significantly higher in the tumor group, whereas osteoblastic changes were significantly higher in the non-tumor group. Using multivariate logistic regression analysis adjusted for seven variables, including age; sex; history of malignancy; change on CT images; the presence of pedicle, the posterior element, and extra-vertebral lesions; history of malignancy (OR 5.0, *p* < 0.01), pedicle lesions (OR 3.5, *p* < 0.05) and extra-vertebral lesions (OR 4.3, *p* < 0.05) were found to be related to tumors; and mixed changes on CT were found to be related to non-tumors (OR 0.1, *p* < 0.05) (Table 6).

In comparison with the malignancy and the non-malignancy groups, the univariate analysis also showed significant differences in CT imaging (*p* < 0.05), pedicle lesions (*p* < 0.01), and extra-vertebral lesions (*p* < 0.01) (Table 7). Multivariate logistic regression analysis adjusted for the seven variables revealed that a history of malignancy (OR: 4.0, *p* < 0.05), pedicle lesions (OR: 5.2, *p* < 0.01), and extravertebral lesions (OR: 3.2, *p* < 0.05) were associated with malignancy (Table 8).

## 4. Discussion

In this study, the diagnostic rate of image-guided percutaneous needle biopsy was as high as 94.0%, and no difference in the diagnostic accuracy between the fluoroscopic and CT-guided biopsies was found. The analysis suggested that the final diagnosis was more likely to be a tumor or malignant tumor if the patient had pedicle lesions, extravertebral lesions, or a history of malignancy.

The diagnostic rate of image-guided percutaneous spinal needle biopsy was reported to be 80–97.3% [7,8,9,10,11,12], which is consistent with the present results. Yang et al. [8] reported a decreased diagnostic rate for lesions <20 mm. However, CT-guided biopsy for small lesions did not result in a decreased diagnostic rate in this present study. Compared with fluoroscopic biopsy, CT-guided biopsy has the advantage that the needle tip could be accurately located. Thus, the diagnostic rate of CT-guided biopsy, even for smaller lesions or difficult cases, was comparable to that of fluoroscopic biopsy in easy cases. Furthermore, in the present case series, skilled spine surgeons decided whether to perform fluoroscopy- or CT-guided biopsies. This may also have influenced the comparatively high diagnostic accuracy of the two methods. With the recent development of molecular-targeted drugs and immune checkpoint inhibitors, biopsies have become increasingly important for determining treatment modalities. Percutaneous needle biopsy is a relatively safe examination with few complications [13] and may be useful when diagnoses are difficult to make using imaging studies.

In this study, 70.9% of the patients exhibited either osteolytic or osteoblastic changes on CT imaging. A higher percentage of the patients in the tumor or malignant group had osteolytic changes, and a higher percentage of patients in the non-malignant group had osteoblastic changes on CT. Tumor cells produce factors that stimulate osteoclast activation and/or osteoblast proliferation [14], which may cause bone destruction and new bone formation. However, clinically, many bone metastases show osteolytic changes, which is consistent with the present prevalence in the malignant group. However, multivariate analysis showed that osteolytic or osteoblastic change was not an independent explanatory factor for tumors or malignancy, whereas mixed change was related to non-tumor status. CT findings were significantly correlated with the prevalence of extravertebral lesions, which might have affected the results. Nevertheless, our results indicated that the absence of osteolytic changes could not rule out malignancy.

A higher percentage of patients in the malignant group had pedicle or extravertebral lesions. Most spinal malignancies can be metastatic tumors, which were also observed in the present study. Metastatic tumors of the spine have been reported to metastasize hematogenously via the paraspinal venous plexus (Batson’s venous plexus), which is characterized by the absence of valve structures [15] and often metastasizes to the posterior side of the vertebral body [16,17]. However, in this present study, the presence of lesions in the vertebral body was not an independent factor related to tumors or malignancy. The winking owl sign [18] is a well-known finding that may indicate metastatic tumors of the vertebral body. Cuénod et al. [19] reported that low signal intensity on MRI T1-weighted images of one or more pedicles was suggestive of malignancy, with a sensitivity of 80% and specificity of 94%. Tumors, specifically malignant ones, often infiltrate the cortical bone and protrude toward low-pressure areas, resulting in extravertebral lesions. Abdel-Wanis et al. [20] reported that an epidural mass or a paraspinal mass was MRI features suggestive of malignant fractures. The results of our study are consistent with these of previous reports and biopsy should be aggressively considered in cases with pedicle and/or extra-vertebral lesions. However, pedicle lesions [21] or posterior cortical protrusion of the vertebral body [19] could also be observed in osteoporotic vertebral fractures, and spinal infections often cause perivertebral or extradural abscesses [22]. Therefore, the decision for biopsy should not be made only based on imaging findings but also comprehensively considering the history of trauma and laboratory findings. 

A high percentage of patients in the tumor and malignancy groups had a history of malignancy. Since most spinal tumors are metastatic, physicians usually ask about the history of the disease when they suspect malignancy at the initial visit. However, whether this medical interview could affect the final diagnosis has rarely been discussed. The results of this study reaffirm the importance of examining medical history. However, of the 43 patients who had a history of malignancy and underwent biopsy, less than half (17 cases, 39.5%) were diagnosed with metastasis from previously diagnosed tumors, and 6 cases were (14.0%) diagnosed with different malignant tumors from previously diagnosed tumors. Specific genetic mutations, such as those in *p53* and *ErbB*, could cause double or triple cancers [23,24]. Although malignancy must be considered first when a patient has a history of malignancy, the possibility of a non-pre-existing malignancy or other pathologies should also be considered when the diagnosis is doubtful, owing to the condition or history of the primary malignant tumor.

This study has several limitations. The first was selection bias. This study only included cases in which a biopsy was performed because of a suspected tumor or malignancy. Therefore, the study population did not include cases in which the examining physician determined that the patient had a disease other than tumor/malignancy, such as an osteoporotic vertebral fracture or pyogenic spondylitis, or cases easily diagnosed as a metastasis from primary cancer. The criteria for biopsy also varied among physicians, and the present results may not be applicable to all cases indicated for biopsy. Second, the sample size was small. The lesion characteristics varied among different tumors. Since the number of cases was too small to analyze each tissue individually, all tumors or malignant tumors were included in the analysis. Therefore, there might be a possibility of bias in imaging features, and further studies with larger study populations are needed to resolve this issue.

In conclusion, the diagnostic rate of image-guided percutaneous needle biopsy was 94.0%. Fluoroscopic- and CT-guided procedures had comparable diagnostic rates. Although the choice of image-guided method must be made based on a case-by-case basis, a needle biopsy is a useful diagnostic examination. In patients with a history of malignancy, pedicle lesions, or extravertebral lesions, the final diagnosis was more likely to be a malignancy. It is suggested that a biopsy should be performed in cases of these characteristics for early diagnosis and subsequent treatment choice. However, follow-up imaging examination instead of biopsy may be selected in patients without these characteristics.

## Figures and Tables

**Table 1:** Demographic characteristics.

*n*	117
Age, mean ± SD	62.5 ± 15.4
Sex (Male), *n* (%)	73 (62.4)
Guiding method, *n* (%)	
Fluoroscopic	73 (62.4)
CT-guided	44 (37.6)
Spinal level, *n* (%)	
Cervical spine	7 (6.0)
Thoracic spine	37 (31.6)
Lumbar spine	57 (48.7)
Sacral spine	16 (13.7)
History of malignancy (+), *n* (%)	43 (36.8)

SD: standard deviation; CT: computed tomography.

**Table 2:** Final diagnosis.

Diagnostic, *n* (%)	110 (94.0)
Non-diagnostic, *n* (%)	7 (6.0)
Tumor, *n* (%)	66 (56.4)
Non-tumor, *n* (%)	51 (43.6)
Malignancy, *n* (%)	56 (47.9)
Non-malignancy, *n* (%)	61 (52.1)
Same as pre-existing tumor, *n* (%)	17 (14.5)
Others, *n* (%)	100 (85.5)

**Table 3:** Comparison of diagnostic accuracy between the fluoroscopic and CT-guided biopsies.

	Diagnostic	Not Diagnostic	*p*-Value
Fluoroscopic, *n* (%)	68 (93.2)	5 (6.8)	
CT-guided, *n* (%)	42 (95.5)	2 (4.5)	0.709

CT: computed tomography.

**Table 4:** Data on imaging features and lesion location.

	n (%)
CT	
Osteolytic change	52 (44.4)
Osteoblastic change	21 (17.9)
Mixed change	10 (8.5)
No change	34 (29.1)
T1-weighted MRI	
Low intensity	111 (94.9)
High intensity	4 (3.4)
Mixed intensity	2 (1.7)
T2-weighted MRI	
Low intensity	70 (59.8)
High intensity	29 (24.8)
Mixed intensity	18 (15.4)
Vertebral body lesion (+)	111 (94.9)
Pedicle lesion (+)	76 (65.0)
Posterior element lesion (+)	43 (36.8)
Extravertebral lesion (+)	61 (52.1)

CT: computed tomography, MRI: magnetic resonance imaging.

**Table 5:** Univariate analysis of the tumor group versus the non-tumor group.

	Tumor	Non-Tumor	*p*-Value
*n*	66	51	
Age, median (IQR)	65.0 (52.3–72.8)	66.0 (57.5–73.0)	0.369
Sex (Male), *n* (%)	37 (56.1)	36 (70.6)	0.157
History of malignancy (+), *n* (%)	25 (37.9)	18 (35.3)	0.925
CT, *n* (%)			
Osteolytic change	40 (60.6)	12 (23.5)	
Osteoblastic change	8 (12.1)	13 (25.5)	
Mixed change	3 (4.5)	7 (13.7)	
No change	15 (22.7)	19 (37.3)	<0.001
T1-weighted MRI, *n* (%)			
Low intensity	63 (95.5)	48 (94.1)	
High intensity	2 (3.0)	2 (3.9)	
Mixed intensity	1 (1.5)	1 (2.0)	1
T2-weighted MRI, *n* (%)			
Low intensity	35 (53.0)	35 (68.6)	
High intensity	20 (30.3)	9 (17.6)	
Mixed intensity	11 (16.7)	7 (13.7)	0.203
Vertebral body lesion (+), *n* (%)	63 (95.5)	48 (94.1)	1
Pedicle lesion (+), *n* (%)	53 (80.3)	23 (45.1)	<0.001
Posterior element lesion (+), *n* (%)	33 (50.0)	10 (19.6)	0.001
Extravertebral lesion (+), *n* (%)	46 (69.7)	15 (29.4)	<0.001

IQR: interquartile range, CT: computed tomography, MRI: magnetic resonance imaging.

**Table 6:** Multivariate analysis of the tumor group versus the non-tumor groups.

	OR	95% CI	*p*-Value
Age	1.0	0.9–1.0	0.062
Sex	0.6	0.2–1.6	0.267
History of malignancy	5.0	1.6–15.8	0.006
CT [Osteolytic change]	2.3	0.7–7.6	0.159
CT [Osteoblastic change]	0.3	0.1–1.2	0.083
CT [Mixed change]	0.1	0.0–0.8	0.028
Pedicle lesion	3.5	1.1–10.8	0.028
Posterior element lesion	2.5	0.7–8.6	0.164
Extravertebral lesion	4.3	1.4–13.3	0.012

CT: computed tomography.

**Table 7:** Univariate analysis of the malignancy group versus the non-malignancy group.

	Malignancy	Non-Malignancy	*p*-Value
*n*	56	61	
Age, median (IQR)	66.5 (56.5–73.0)	64.0 (53.0–73.0)	0.490
Sex (Male), *n* (%)	33 (58.9)	40 (65.6)	0.582
History of malignancy (+), *n* (%)	23 (41.1)	20 (32.8)	0.461
CT, *n* (%)			
Osteolytic change	33 (58.9)	19 (31.1)	
Osteoblastic change	7 (12.5)	14 (23.0)	
Mixed change	3 (5.4)	7 (11.5)	
No change	13 (23.2)	21 (34.4)	0.025
T1-weighted MRI, *n* (%)			
Low intensity	55 (98.2)	56 (91.8)	
High intensity	1 (1.8)	3 (4.9)	
Mixed intensity	0 (0)	2 (3.3)	0.368
T2-weighted MRI, *n* (%)			
Low intensity	32 (57.1)	38 (62.3)	
High intensity	16 (28.6)	13 (21.3)	
Mixed intensity	8 (14.3)	10 (16.4)	0.659
Vertebral body lesion (+), *n* (%)	55 (98.2)	56 (91.8)	0.209
Pedicle lesion (+), *n* (%)	47 (83.9)	29 (47.5)	<0.001
Posterior element lesion (+), *n* (%)	26 (46.4)	17 (27.9)	0.059
Extravertebral lesion (+), *n* (%)	39 (69.6)	22 (36.1)	<0.001

IQR: interquartile range, CT: computed tomography, MRI: magnetic resonance imaging.

**Table 8:** Multivariate analysis of the malignancy group versus the non-malignancy groups.

	OR	95% CI	*p*-Value
Age	1.0	1.0–1.0	0.428
Sex	0.8	0.3–2.2	0.724
History of malignancy	4.0	1.4–11.6	0.011
CT [Osteolytic change]	2.1	0.7–6.3	0.193
CT [Osteoblastic change]	0.6	0.1–2.3	0.418
CT [Mixed change]	0.4	0.1–2.3	0.306
Pedicle lesion	5.2	1.8–15.6	0.003
Posterior element lesion	1.0	0.3–3.2	0.944
Extravertebral lesion	3.2	1.1–9.3	0.036

CT: computed tomography.

## Data Availability

The data that support the findings of this study are available from the corresponding author, A.S., upon reasonable request.
